# Insight and cognitive complaints in stabilized outpatients with schizophrenia

**DOI:** 10.1192/j.eurpsy.2024.1574

**Published:** 2024-08-27

**Authors:** S. Ajmi, M. Bouhamed, R. Ouali, S. Hentati, I. Feki, R. Sallemi, J. Masmoudi

**Affiliations:** Psychiatrie A, HEDI CHAKER University Hospital, SFAX, Tunisia

## Abstract

**Introduction:**

Schizophrenia is often considered as pathology of consciousness. Some authors have considered that patients’ self-perception of their cognitive difficulties expressed in the form of subjective complaints could represent a source of stress. These cognitive difficulties may then interfere with the interpretation of symptoms, leading to poor insight.

Insight and cognitive complaints in stabilized outpatients with schizophrenia.

**Objectives:**

Study the relationship between subjective cognitive complaints and clinical insight in a Tunisian population with schizophrenia.

**Methods:**

This is a cross-sectional, descriptive and analytical study carried out on 72 stabilized patients followed at the post-cure psychiatry consultation ‘A’ at the CHU Hédi Chaker in Sfax diagnosed with schizophrenia according to the DSM 5 criteria.

We used the schedule for the Assessment of Insight–Expanded Version(SAI-E) scale to assess Clinical Insight and the Subjective Scale to Investigate Cognition in Schizophrenia (SSTICS) to determine subjective cognitive complaints

**Results:**

The mean age of the patients was 46.83 ± 11.6 years, with a sex ratio (M/F) of 2. In our study, 48.5% were single and 69.4% were unemployed.

The median total SSTICS score was 25.

Using the SAI-E scale, an average score of 20.1 was objectified in our study.

In our study, the better the insight, the greater the subjective cognitive complaints were in all cognitive domains (p=0.00).

Awareness of illness was statistically associated with working memory (p=0.001), explicit memory (p=0.004), attention (p=0.001), language (p=0.01) and executive functions (p=0.001).

**Conclusions:**

Our study highlights the relationship between awareness of illness and cognitive complaints. The clinician, faced with repetitive cognitive complaints, should assess the insight before incriminating another cause (effects of a drug, cognitive deficit, etc.).

**Disclosure of Interest:**

None Declared

